# Supplementary material for: Does Dosage Drive Durability? A Longitudinal Study on Digital Cognitive Training for Sustained Functional Independence
Source: Geriatr Gerontol Int. 2026 Sep 25;26(10):e70848. doi: 10.1111/ggi.70848 (PMC13614873; doi:10.1111/ggi.70848)
Supplement: Supplementary file 1 [file GGI-26-0-s001.pdf]

International Journal of Geriatric Psychiatry

Research Article

Does Dosage Drive Durability? A Longitudinal Study on Digital Cognitive Training for Sustained Functional Independence

Submission ID a2353001-e4fd-4dac-bdfa-bafadc7ac06e

Submission Version Initial Submission

PDF Generation 16 Feb 2026 21:57:49 EST by Atypon ReX

Authors

Dr. Bruno Costa Poltronieri
Submitting Author

ORCiD
https://orcid.org/0000-0002-2127-0574

Affiliations
- Instituto de Ciências Biomédicas, Universidade Federal do Rio de Janeiro, Rio de Janeiro, Brazil
- Instituto de Psiquiatria, Universidade Federal do Rio de Janeiro, Rio de Janeiro, Brazil
- Instituto Federal de Educação, Ciência e Tecnologia do Rio de Janeiro, Rio de Janeiro, Brazil

Dr. Cíntia Monteiro Carvalho

ORCiD
https://orcid.org/0000-0002-8115-7426

Affiliations
- Instituto de Ciências Biomédicas, Universidade Federal do Rio de Janeiro, Rio de Janeiro, Brazil
- Instituto de Psiquiatria, Universidade Federal do Rio de Janeiro, Rio de Janeiro, Brazil

Ms. Karin Reuwsaat

ORCiD
https://orcid.org/0000-0001-5061-3591

Affiliations
- Instituto de Ciências Biomédicas, Universidade Federal do Rio de Janeiro, Rio de Janeiro, Brazil
- Instituto de Psiquiatria, Universidade Federal do Rio de Janeiro, Rio de Janeiro, Brazil

Miss Maria Eduarda Alves Reis

Affiliations
- Instituto de Ciências Biomédicas, Universidade Federal do Rio de Janeiro, Rio de Janeiro, Brazil
- Instituto de Psiquiatria, Universidade Federal do Rio de Janeiro, Rio de Janeiro, Brazil

Miss Ana Carolina Madeira

Affiliations

- Instituto de Ciências Biomédicas, Universidade Federal do Rio de Janeiro, Rio de Janeiro, Brazil
- Instituto de Psiquiatria, Universidade Federal do Rio de Janeiro, Rio de Janeiro, Brazil

Miss Thayná de Carvalho Freire

Affiliations

- Instituto de Psiquiatria, Universidade Federal do Rio de Janeiro, Rio de Janeiro, Brazil
- Instituto Federal de Educação, Ciência e Tecnologia do Rio de Janeiro, Rio de Janeiro, Brazil

Dr. Rogerio Panizzutti
Corresponding Author

Affiliations

- Instituto de Ciências Biomédicas, Universidade Federal do Rio de Janeiro, Rio de Janeiro, Brazil
- Instituto de Psiquiatria, Universidade Federal do Rio de Janeiro, Rio de Janeiro, Brazil

Files for peer review

All files submitted by the author for peer review are listed below. Files that could not be converted to PDF are indicated; reviewers are able to access them online.

Name	Type of File	Size	Page
Main text IJGP (1).docx	Main Document - MS Word	5.0 MB	Page 4
FIGURE 1.FU.jpg	Figure	250.5 KB	Page 27
table.docx	Table	51.0 KB	Page 28

Does Dosage Drive Durability? A Longitudinal Study on Digital Cognitive Training for Sustained Functional Independence

Bruno Costa Poltronieri[1,2,3],PhD, Cíntia Monteiro Carvalho[1,2],PhD, Karin Reuwsaat[1,2],BS, Maria Eduarda Alves Reis[1,2],BS, Ana Carolina Madeira[1,2], college student, Thayná de Carvalho Freire [2, 3], college student, Rogerio Panizzutti[1,2], MD,PhD

[1] Instituto de Ciências Biomédicas, Universidade Federal do Rio de Janeiro, Rio de Janeiro, Brazil
[2] Instituto de Psiquiatria, Universidade Federal do Rio de Janeiro, Rio de Janeiro, Brazil
[3] Instituto Federal de Educação, Ciência e Tecnologia do Rio de Janeiro, Rio de Janeiro, Brazil

Address correspondence: Rogério Panizzutti. Instituto de Psiquiatria, Universidade Federal do Rio de Janeiro. Avenida Venceslau Brás 71, Rio de Janeiro, RJ 22290140, Brazil. E-mail: rogerio.panizzutti@ipub.ufrj.br. Tel.: +55 (21) 3938-5588.

Key points

- This clinical trial investigated whether a higher cumulative dose of Digital Cognitive Training (DCT) leads to greater and more sustained improvements in cognition and functionality among older adults with Mild Neurocognitive Disorder.
- Only the group that received 20 hours of DCT maintained significant gains in functionality at the 3 and 6-month follow-up.

- Both intervention doses (10h and 20h of DCT) resulted in cognitive improvements immediately after training, specifically in domains such as processing speed and recognition.

Acknowledgements

We are grateful to the participants for their involvement in this study.

Funding

This work was supported by the National Institutes of Health – Fogarty International Center (Grant R03TW009002 to RP); Fundação de Amparo à Pesquisa do Estado do Rio de Janeiro (FAPERJ) (Grant E-26/ 110.305/2014 to RP) and Conselho Nacional de Desenvolvimento Científico e Tecnológico (CNPq) (Grant 400455/2012-9 to RP). Posit Sciences provided the exercises of the intervention groups at no cost. The authors of the current manuscript declare that the funding received from such agencies did not limit the ability to complete the investigation as planned and publish the results. They had full control of all primary data. We also declare that we have the data available so that Archives of Gerontology and Geriatrics can review it, if requested.

Ethics Statement

The research was approved by the Ethics Committee of the Federal University of Rio de Janeiro - Institute of Psychiatry (4.315.008)

Consent

All participants gave informed written consent prior to study inclusion for their information to be used for research, and were treated in accordance with the declaration of Helsinki.

Data availability and clinical trial registration

The data are available upon request to the researcher for replication purposes, including access to analytic methods and materials. The studies reported in the manuscript were pre-registered at ClinicalTrials.gov under the identifier NCT03911765.

Declaration of competing interest

R.P. is the founder of NeuroForma LTDA, a company with a financial interest in cognitive training. The remaining authors have no conflict of interest to disclose.

CRediT authorship contribution statement

Bruno Costa Poltronieri: Writing – original draft, Visualization, Supervision, Resources, Project administration, Investigation, Formal analysis, Data curation, Conceptualization.

Cintia: Visualization, Supervision, Software, Resources, Project administration, Investigation, Formal analysis, Data curation, Conceptualization.

Karin: Visualization, Resources, Investigation, Data curation, Conceptualization.

Maria Eduarda: Visualization, Software, Resources, Project administration.

Ana Carolina: Visualization, Software, Resources.

Thayná Freire: Visualization, Software, Resources.

Rogério Panizzutti: Writing – review & editing, Visualization, Supervision, Resources, Project administration, Methodology, Conceptualization.

Abstract

Background: This study aimed to investigate whether a higher cumulative dose of digital cognitive training leads to greater and more sustained improvements in cognition and functionality among older adults with Mild Neurocognitive Disorder.

Methods: We recruited participants aged 60 or older with mild neurocognitive disorder to a double-blinded, randomized, stepped wedge clinical trial of digital cognitive training (DCT) compared to an active control group of commercial computer games. Participants were evaluated for functionality and cognition before, after 10h and 20h of intervention and in the follow up.

Results: Sixty-seven participants completed DCT and were evaluated. The group that received 20h of DCT showed significant improvements in functionality and satisfaction, as well as gains in processing speed, verbal fluency, and inhibitory control. The 10h group also showed improvements in specific areas such as processing speed and recognition. In the 20h group, the benefits were maintained at 3- and 6-month follow-ups, especially in functionality.

Discussion: This study suggests that a higher cumulative dose of digital cognitive training may lead to more sustained improvements in functionality performance among older adults with mild neurocognitive disorder. Findings

highlight the need for further research to determine optimal training dosage for long-term improvements in cognition and functionality.

Abstract word count (200 words)

Keywords: mild neurocognitive Disorder, cognitive training, functionality, brain plasticity

Introduction

Global population aging presents major health and social challenges, especially regarding cognitive decline from normal aging to dementia.[1] Mild Neurocognitive Disorder is defined as an intermediate stage between normal cognitive aging and dementia, involving deficits in specific cognitive domains without impairment of daily activities.[2-4]

Although mild neurocognitive disorder does not significantly impair overall functionality, older adults with this condition may experience difficulties performing instrumental activities of daily living.[5] Research has shown a strong association between executive functions and daily functioning.[6,7] These findings highlight the importance of developing and investing in interventions, such as DCT, that support and enhance executive functions.

DCT has been increasingly explored as an intervention that can mitigate cognitive decline in older adults, such as mild neurocognitive disorder. DCT encompasses structured practice with standardized and cognitively demanding tasks[8] and provides various benefits, such as engaging visual interfaces, efficient and scalable implementation, and the capacity to dynamically adjust training content and difficulty based on individual performance.[9,10] Meta-analysis on DCT in older adults with mild neurocognitive disorder have reported effects ranging

from small to moderate for global cognition, working memory, memory, and learning.[11,12]

Despite these promising results, some challenges remain unclear regarding DCT, including the optimal cumulative dose and the duration of cognitive effects in older adults with mild neurocognitive disorder. Evidence suggests that sessions shorter than 30 minutes are ineffective[10], while around 50–55 minutes, six times per week, may yield better outcomes[13]. However, it is still uncertain whether higher cumulative doses produce greater benefits or if the effects persist long term, as most studies assess only immediate outcomes[10,11,] Some research, however, shows sustained benefits, such as global cognition improvement 3 months post-training[14] and maintained episodic memory gains 6 months later.[15] Yet, neither study examined the long-term impact of different cumulative doses of DCT in older adults with mild neurocognitive disorder.

Recently, our group conducted a stepped wedge randomized clinical trial in which we explored the effects of DCT exercises primarily focused on enhancing executive functions on functionality and cognition in older adults with mild neurocognitive disorder.[16] To assess functionality, we employed the Canadian Occupational Performance Measure (COPM), an individualized tool widely used in rehabilitation settings across several countries, designed to identify difficulties in activities of daily living and provide a comprehensive understanding of individual challenges. Participants who underwent 10h of DCT showed significant improvements in COPM scores and learning compared to those who engaged in leisure games.[16]

Building on these findings, the present study examines the impact of 10 or 20h of DCT on functionality and cognition, immediately after training and 3 and 6

months after the end of training. We pose the following questions: 1) Are there significant differences in the effects of 10 or 20h of DCT on functionality and cognition after training? 2) Are there significant differences in the effects of 10h or 20h of DCT on functionality and cognition at 3 and 6-months follow-ups?

Methods

Participants

Participants were recruited through professional referrals and social media advertisements and contacted the research team via email or text message. Eligibility criteria for inclusion in the study were: age ≥ 60 years; normal or corrected vision and hearing; access to the internet; and diagnosis of Mild Neurocognitive Disorder based on DSM-5 criteria. Exclusion criteria included: a diagnosis of dementia; dependence on activities of daily living; and major medical conditions that precluded study participation.

All participants underwent a thorough evaluation, which included clinical history, cognitive and functional assessments, and evaluations of anxiety and depression symptoms. The clinical diagnosis was established in accordance with DSM-5 criteria and confirmed by a board-certified psychiatrist (R.P.).

Participants were assessed during the study period from July 2020 to January 2025. Before enrollment, all participants provided written informed consent after receiving a detailed explanation of the study procedures. The research was approved by the Ethics Committee of the Federal University of Rio

de Janeiro - Institute of Psychiatry (4.315.008) and pre-registered at ClinicalTrials.gov (NCT03911765).

Study Design

The study was a randomized, double-blinded, stepped wedge clinical trial.[17] Participants were randomized by an independent researcher using a hierarchical stratification by age, education, MoCA score, gender, Technology activities of daily living questionnaire (TADLQ), and randomly assigned to one of the groups. Initially, only one group performed 10h of DCT and the other group played 10h of commercial games. Then, both groups performed an additional 10h of DCT. Thus, one group performed a total of 10h of DCT and the other performed 20h of DCT. We performed the follow up assessments three and six months after the end of DCT.

All recruited participants (n=67) were assessed for eligibility, and those who met the inclusion criteria underwent cognitive and functional assessments before starting the training, immediately after 10h and 20h of training, three and six months after the training for follow-up. The assessment battery was applied by a team of clinicians composed of occupational therapists and neuropsychologists. Participants did not receive any financial incentive to participate in the study.

Participants trained at home either on their own or on a study-provided laptop or tablet. They were asked to train as much as possible, one hour per session 2 twice a week. Subjects in both groups were remotely monitored via video call by a designated research assistant, who provided support with issues such as technical issues or adherence to the training protocol.

Measurements

An assessment battery was administered to measure participants' cognition and functionality. Regarding cognitive domains, we investigated verbal fluency, memory (including long-term memory, short-term memory, and recognition), learning over trials, processing speed, cognitive flexibility, and global cognition. To this end, the following instruments were used: the Montreal Cognitive Assessment (MoCA)[16,18] to assess global cognition; the Verbal Fluency Test to assess verbal fluency;[19] the Rey's Auditory-Verbal Learning Test (RAVLT)[20] to evaluate long-term memory (list A7—number of words recalled after a 20- to 30-minute delay), short-term memory (list A6—number of words recalled after the interference list, with scores ranging from 0 to 15), recognition (list A and non-list A words correctly identified), and learning over trials (sum of words recalled across five trials minus five times the first trial, with scores ranging from 0 to 75); and the Stroop Test[21] to measure processing speed and inhibitory control, referring to card 1 and card 3, respectively.

To evaluate functionality, we applied the Canadian Occupational Performance Measure (COPM)[22] and the Technology Activities of Daily Living Questionnaire (TADLQ).[23] The COPM is a semi-structured, client-centered interview designed to assess participants' self-perceived occupational performance and satisfaction in relation to specific individualized functional challenges,[24] whereas the TADLQ is a structured questionnaire that evaluates the functionality of older adults to perform daily activities, including a specific subscale for technology use.[23]

To assess neuropsychiatric symptoms, we applied the Geriatric Depression Scale 15 (GDS)[25] and Geriatric Anxiety Inventory (GAI).[26]

Cognitive training exercises

The current study was based on the hypothesis that training with digital exercises focused on executive functions could improve the functionality of the participants.[16] Thus, we designed the digital neuroscience-informed cognitive training using a series of exercises from the BrainHQ platform (www.brainhq.com, PositScience, Inc.) focused on improving executive functions. The exercises were: Divided Attention, Eye For Detail, Target Tracker, Juggle Factor, Double Decision, Right Turn, Mental Map, and Optic Flow.

The 10h cognitive training group began the first phase of the study by engaging in computer-based leisure games, including *The Birds Game*, *Let's Clean Up*, *Bubble Poke*, *Word Search*, *Memory Game*, *Puzzle*, *Dominoes*, *Smarty Bubbles*, and *Spot the Difference*. After completing 10h of these leisure games, participants proceeded to a second phase consisting of 10h of DCT. The 20h cognitive training group completed the cognitive exercises throughout the study, practicing each exercise for 15 minutes per session until completing 1 hour of DCT per session. Full details about the group conditions are provided in Carvalho et al.[16]

Statistical Analysis

We verified data normality using the Shapiro–Wilk test. Winsorized means were calculated for verbal fluency, GDS, TADLQ, processing speed, inhibitory control, and learning over trials and constituted 10% of the total data. Measures were normally distributed after winsorizing of outlying values. We used T-tests and chi-square tests to compare sociodemographic characteristics, functionality, cognition and neuropsychiatric symptoms between groups at baseline. We used a mixed-model analysis of covariance (ANCOVA) to determine the effects of DCT, using baseline scores and education as covariates. Post hoc analyses used

one-way repeated-measures analysis of variance (RM-ANOVA), with Sidak corrections to adjust for multiple comparisons. Time (pre-training, post-training, 3-month follow-up, and 6-month follow-up) was treated as the intra-group factor, and training dose as the inter-group factor. Spearman's rho correlation was used to assess the association between cognition and functionality. Only participants who completed all study phases were included in the analysis. We used SPSS version 23 for statistical analyses. Cohen's d values were derived from eta squared.

Results

Participants

We assessed 138 participants and excluded 51 because of ineligibility or lack of interest in the study. Eighty-seven participants were randomly assigned to one of the two groups: 10h of DCT (n=45) or 20h of DCT (n=42). Of the total number of randomized participants, 67 completed the protocol of DCT, while 41 were evaluated 3 months post-training of DCT, and 31 were evaluated 6 months post-training. Post-training attrition rates were 24.4% in the 10h DCT group and 21.4% in the 20h of DCT group. At the 3-month follow-up, attrition increased to 38.3% in the 10h group and 39.3% in the 20h group. By the 6-month follow-up, attrition rates rose further to 53% for the 10h group and 54.5% for the 20h group. Participants were predominantly composed of women with 14 years of education (SD=5.2) and a mean age of 74.9 (SD=7.2). There were no significant sociodemographic and clinical differences between the two randomized groups (Table 1).

Changes in functionality and cognition post-training

Mixed-model ANCOVA using the baseline scores and education as covariates revealed a global effect over time post-training in COPM performance (F (1,54) = 6.18, p=0.016, d=0.67) and in COPM satisfaction (F (1,51) = 9.21, p=0.004, d=0.85) with no significant group-by-time interaction for both groups. Post hoc one-way RM-ANOVA for the two groups separately revealed significant improvement over time in COPM performance to 20h of DCT group (F (1,22) = 14.5, p=0.001, d=1.62) and also to COPM satisfaction (F (1,24)=12.05, p=0.002, d=1.41). There was no change in COPM Performance and COPM satisfaction for 10h DCT group (Image 1). Additionally, there was no effect on the TADLQ measure of functionality for 20h of DCT group, without effect over time for both groups (Table 2).

The analysis of individual cognitive domains, controlling for multiple comparisons, showed a significant effect of time for both groups over 20 h of training for verbal fluency, learning over trial, recognition, processing speed, inhibitory control and short term memory (Table 2). Post hoc analyses demonstrated improvements over time in specific domains for the 10h DCT group, including processing speed (F(1,31) = 14.81, p = 0.001, d = 1.38), recognition (F(1,25) = 33.44, p < 0.001, d = 2.31), learning over trials (F(1,25) = 9.74, p = 0.004, d = 1.24), and inhibitory control (F(1,31) = 10.01, p = 0.003, d = 1.13).

Similarly, the 20h of DCT group showed significant improvements over time in processing speed (F (1,29) = 3.31, p = 0.04, d= 0.67), recognition (F (1,26) = 15.23, p = 0.001, d = 1.52), learning over trials (F (1,26) = 14.68, p = 0.001, d= 1.50), and verbal fluency (F(1,30) = 4.97, p = 0.03, d = 0.81). However, no significant effect on short-term memory was observed for either group following the post hoc analyses. Finally, we conducted a Pearson correlation to identify which changes in cognitive domains were associated with improvement on the

COPM. A significant correlation was observed between this variable and verbal fluency (r = 0.39; p = 0.02) in the group that underwent 20h of DCT.

Changes in functionality and cognition in the follow-up

In order to examine the durability of the effects, we performed a follow-up assessment after 3 months (Table 3) and 6 months (Table 4) of the end of training sessions. The follow-up assessment revealed a main effect of time in COPM performance for the 20h of DCT group after 3 months (F (1,14) = 14.0, p=0.001, d=2.37) with a significant group-by-time interaction (F (1,33) = 14.11, p = 0.001, d=1.03). These results persisted until 6 months after the end of the training program, with improvements over time (F (1,25) = 29.07, p<0.001, d=1.32) with a significant group-by-time interaction (F(1,25)=9.03, p=0.006,d=1.20) for 20h of DCT group.

With regard to COPM Satisfaction, the post hoc analyses showed improvements in COPM satisfaction for 20h of DCT group F(1,12)=18.35, p<0.001, d=2.47) without effects for 10h of DCT group. However, in the assessment performed after 6 months, the COPM satisfaction improved significantly over time (F(1,10)=10.92, p=0.008, d=2.09) for 20h of DCT group. There was no change in COPM Performance and COPM satisfaction in 10h of DCT group in follow-up (Figure 1).

When analyzing individual cognitive domains, no group-by-time interaction was observed in follow-up. However, there was a significant effect of time in processing speed for 10h of DCT (F(1,17) = 6.200, p=0.02, d = 1.20) and 20h of DCT group (F(1,18) = 20.70, p = 0.001, d = 2.14). These improvements in processing speed persisted at the 6-month follow-up assessment for both the 10h DCT group (F(1,12) = 9.01, p = 0.01, d = 1.73) and the 20h of DCT group (F(1,12) = 6.64, p = 0.02, d = 1.48).

Regarding inhibitory control at the 3-month follow-up, the 10h DCT group showed a trend toward improvement in inhibitory control ($F(1,17) = 3.68$, $p = 0.07$, $d = 0.93$), while the 20h of DCT group demonstrated a significant improvement over time ($F(1,18) = 5.92$, $p = 0.02$, $d = 1.14$). At the 6-month follow-up, both groups exhibited significant improvements: the 10h DCT group ($F(1,12) = 5.09$, $p = 0.04$, $d = 1.30$) and the 20h of DCT group ($F(1,12) = 14.17$, $p = 0.003$, $d = 2.17$).

For recognition, the 10h DCT group showed significant improvement at the 3-month follow-up ($F(1,18) = 35.74$, $p < 0.001$, $d = 2.81$), while the 20h of DCT group did not ($F(1,17) = 0.75$, $p = 0.39$, $d = 0.04$). At the 6-month follow-up, the 10h DCT group maintained significant improvement ($F(1,12) = 7.50$, $p = 0.01$, $d = 1.58$), whereas the 20h of DCT group did not reach significance ($F(1,12) = 2.44$, $p = 0.14$, $d = 0.90$).

Finally regarding learning over trials, at the 3-month follow-up, both the 10h DCT group ($F(1,14) = 15.07$, $p = 0.002$, $d = 2.07$) and the 20h of DCT group ($F(1,13) = 5.44$, $p = 0.03$, $d = 1.29$) demonstrated significant improvements, however at 6-month follow-up there is no significant effects over time for the both groups.

Discussion

This study investigated the dose-cumulative effects of 10 and 20 hours of DCT on functionality and cognition in older adults with mild neurocognitive disorder, and found that both groups improved cognitively after training; however, only the 20h of DCT group maintained significant functional gains up to six months later, supporting our initial hypothesis[16] that a higher training dose leads to more durable cognitive and functional benefits.

Our findings are consistent with those reported in ACTIVE study,[27] where, after ten years, participants who performed DCT (in three intervention groups) reported fewer difficulties in performing instrumental activities of daily living compared to those in the control group. However, it is important to note that the result in the ACTIVE study was only observed between years five and ten, which differs from our study due to its shorter follow-up period. Unlike our findings, other studies that investigated functionality in older adults with mild neurocognitive disorder by scale or questionnaire to measure daily functioning following cognitive remediation, which includes DCT, virtual reality interventions or neuropsychological rehabilitation, did not report significant changes in this variable.[28-30] There are several possible explanations for the sustained effects of training on self-reported daily functioning, as measured by COPM performance and satisfaction. Our DCT protocol primarily targeted executive function, a broad cognitive domain encompassing initiation, sequencing, monitoring, inhibition, set-shifting, planning, problem-solving,[31] verbal fluency, and other components.[32] Many of these subdomains are moderately associated with performance in daily functioning.[5] Our previous study[16] found that improvements in COPM performance were associated with gains in processing speed and verbal

fluency—both of which showed significant long-term effects in the 20h of DCT group. In the present study, however, we found an association only with change in verbal fluency. The maintenance of COPM-related effects may also be explained by the behavioral changes induced by the cognitive training. The cognitive improvements may have encouraged participants to re-engage in activities they were previously interested in or motivated them to explore new occupations.[27] Regarding the instrument employed to evaluate functionality, studies evaluating the validity of the COPM in different contexts have shown that this instrument demonstrates consistent content validity, construct validity, and feasibility,[33] suggesting that it is a useful tool for assessing daily function. Furthermore, it addresses self-perceived problems that may not be captured by other standardized measures.[34]

The cognitive improvements observed following training occurred irrespective of whether participants received 10 or 20 hours of intervention. This finding is in line with prior research indicating that both short- and long-duration training protocols tend to produce comparable outcomes in terms of near transfer effects; however, extended protocols may enhance certain aspects of far transfer effects.[35] A study published by,[10] which investigated cognitive outcomes before, during, and after multi-domain DCT, revealed a marked therapeutic response, with notable improvements even after a limited number of training sessions. Similarly, previous research has shown acute brain changes following eight weeks of cognitive training,[36] although evidence for the long-term persistence of these changes remains limited.

Despite these encouraging findings, the relationship between dose-cumulative training and cognitive outcomes remains a subject of debate in the literature. A

meta-analysis reported that a lower total number of sessions was associated with more significant long-term effects,[37] which contrasts with another study involving individuals with schizophrenia, suggesting that higher doses of DCT may be necessary to promote lasting cognitive gains.[38] This discrepancy reinforces the notion that the optimal dosage of DCT is not yet well-established, particularly regarding its long-term impact.[39] Considering that cognitive gains do not appear to follow a linear trajectory, it is essential for future research to further investigate the role of dose-cumulative training and explore how maintenance training may influence outcomes over time.

In addition to behavioral outcomes, numerous studies have investigated the neurobiological mechanisms underlying the cognitive improvements associated with cognitive training. DCT has the potential to promote neuroplasticity by engaging the brain in targeted cognitive activities that enhance its functional capacity,[40] a process that may have occurred among the participants in the present study. The effect of neuroplasticity induced by DCT was observed in studies using the diffusion tensor imaging technique. These studies have demonstrated that skills training over several weeks promotes microstructural changes of limbic system structures, reflecting changes in rearrangement of neural tissue.[41,42] Additionally, memory training has been linked to increased activation and connectivity in various brain regions, such as the frontal, temporal, and occipital lobes, as well as the hippocampus, in both cognitively healthy older adults and those with mild neurocognitive disorder. However, the exact extent of these changes in individuals is still not fully understood.[43-46]

As far as we know, this is one of the few studies to examine the cumulative dose of DCT using a stepped-wedge clinical trial design in older adults with mild neurocognitive disorder, with follow-up assessments conducted in the absence of additional training. However, this study has several limitations: 1) As in most follow-up studies, we experienced a significant attrition rate at the six-month assessment, which reduced the statistical power of our sample during that phase; 2) Since the majority of this study was conducted during the COVID-19 pandemic, most participants recruited were older adults with access to technology and the internet. This group is not representative of the broader Brazilian population. To mitigate this limitation, we provided tablets to participants residing in Rio de Janeiro and offered support via videoconference; 3) Although our functionality measures are sensitive and cover diverse constructs, they rely on self-report and self-perception. It is important for future research to also incorporate ecologically valid and performance-based assessment tools, which may offer better insight into the transfer of cognitive training gains to everyday functioning in older adults exposed to varying doses of DCT; 4) Finally, it is important to highlight that due to the high attrition rate during follow-up, it was not feasible to perform an intention to treat analysis, although the authors acknowledge the importance of such analyses in randomized clinical trials.

Although definitive conclusions cannot be drawn, it is possible that the cumulative dose of cognitive training contributed to the sustained effects observed, particularly in the COPM outcomes in our study. Future research should further explore the ideal training dosage, so that clinicians can implement DCT programs with greater precision and effectiveness for older adults with mild neurocognitive disorder.

References

1. Rudnicka E, Napierała P, Podfigurna A, Męczekalski B, Smolarczyk R, Grymowicz M. The world health organization (WHO) approach to healthy ageing. Maturitas 2020;139:6-11. Doi: 10.1016/j.maturitas.2020.05.018.

2. Petersen RC. Mild Cognitive Impairment. Continuum (Minneap Minn) 2016;22(2, Dementia):404-418. Doi: 10.1212/CON.0000000000000313.

3. Petersen RC, Lopez O, Armstrong MJ, et al. Practice guideline update summary: Mild cognitive impairment. Neurology 2018;90(3):126-135. Doi: 10.1212/WNL.0000000000004826.

4. Jongsiriyanyong S, Limpawattana P. Mild Cognitive Impairment in Clinical Practice: A Review Article. Am J Alzheimers Dis Other Demen 2018;33(8):500-507. Doi: 10.1177/1533317518791401.

5. Raimo S, Maggi G, Ilardi CR, et al. The relation between cognitive functioning and activities of daily living in normal aging, mild cognitive impairment, and dementia: a meta-analysis. J Neurol Sci 2024;45(4). Doi: 10.1007/s10072-024-07366-2.

6. Marshall GA, Rentz DM, Frey MT, Locascio JJ, Johnson KA, Sperling RA. Executive function and instrumental activities of daily living in mild cognitive impairment and Alzheimer's disease. Alzheimers Dement 2011;7(3):300-308. Doi: 10.1016/j.jalz.2010.04.005.

7. Martyr A, Clare L. Executive Function and Activities of Daily Living in Alzheimer's Disease: A Correlational Meta-Analysis. Dement Geriatr Cogn Disord 2012;33(2-3):189-203. Doi: 10.1159/000338233.

8. Harvey PD, McGurk SR, Mahncke H, Wykes T. Controversies in Computerized Cognitive Training. Biol Psychiatry Cogn Neurosci Neuroimaging 2018;3(11):907-915. Doi: 10.1016/j.bpsc.2018.06.008.

9. Woodruff E, Poltronieri BC, Sousa LP, et al. Effects of bottom-up versus top-down digital cognitive training in older adults: A randomized controlled trial. Arch Gerontol Geriatr 2024;105552. Doi: 10.1016/j.archger.2024.105552.

10. Lampit A, Hallock H, Moss R, et al. The Timecourse of Global Cognitive Gains from Supervised Computer-Assisted Cognitive Training: A Randomised, Active-Controlled Trial in Elderly with Multiple Dementia Risk Factors. J Prev Alzheimers Dis 2014;1:1-7. Doi: 10.14283/jpad.2014.18.

11. Zhang H, Huntley J, Bhome R, et al. Effect of computerised cognitive training on cognitive outcomes in mild cognitive impairment: a systematic

review and meta-analysis. BMJ Open 2019;9(8):e027062. Doi: 10.1136/bmjopen-2018-027062.

12. Hill NTM, Mowszowski L, Naismith SL, Chadwick VL, Valenzuela M, Lampit A. Computerized Cognitive Training in Older Adults With Mild Cognitive Impairment or Dementia: A Systematic Review and Meta-Analysis. Am J Psychiatry 2017;174(4):329-340. Doi: 10.1176/appi.ajp.2016.16030360.

13. Liu L, Wang H, Xing Y, et al. Dose–response relationship between computerized cognitive training and cognitive improvement. NPJ Digit Med 2024;7(1):64. Doi: 10.1038/s41746-024-01210-9.

14. Hyer L, Scott C, Atkinson MM, et al. Cognitive Training Program to Improve Working Memory in Older Adults with MCI. Clin Gerontol 2015;39(5):410-427. Doi: 10.1080/07317115.2015.1120257.

15. Herrera C, Chambon C, Michel BF, Paban V, Alescio-Lautier B. Positive effects of computer-based cognitive training in adults with mild cognitive impairment. Neuropsychologia 2012;50(8):1871-1881. Doi: 10.1016/j.neuropsychologia.2012.04.012.

16. Carvalho CM, Poltronieri BC, Reuwsaat K, Reis MEA, Panizzutti R. Digital cognitive training for functionality in mild cognitive impairment: a randomized controlled clinical trial. GeroScience 2025. Doi: 10.1007/s11357-024-01464-x.

17. Copas AJ, Lewis JJ, Thompson JA, Davey C, Baio G, Hargreaves JR. Designing a stepped wedge trial: three main designs, carryover effects and randomisation approaches. Trials 2015;16:352. Doi: 10.1186/s13063-015-0842-7.

18. Nasreddine ZS, Phillips NA, Bédirian V, et al. The Montreal Cognitive Assessment, MoCA: A Brief Screening Tool For Mild Cognitive Impairment. J Am Geriatr Soc 2005;53(4):695-699. Doi: 10.1111/j.1532-5415.2005.53221.x.

19. Lezak MD, Howieson DB, Loring DW. Neuropsychological Assessment. 4th ed. Oxford University Press, 2004.

20. Spreen O, Strauss E. *A* Compendium of Neuropsychological Tests: Administration, Norms and Commentary. 2nd ed. Oxford University Press, 1998.

21. Golden CJ. Stroop Color and Word Test: A Manual for Clinical and Experimental Uses. Stoelting Company, 1978.

22. Law M, Baptiste S, McColl M, Opzoomer A, Polatajko H, Pollock N. The Canadian Occupational Performance Measure: An Outcome Measure for

Occupational Therapy. Can J Occup Ther 1990;57(2):82-87. Doi: 10.1177/000841749005700207.

23. Muñoz-Neira C, López OL, Riveros R, Núñez-Huasaf J, Flores P, Slachevsky A. The Technology – Activities of Daily Living Questionnaire: A Version with a Technology-Related Subscale. Dement Geriatr Cogn Disord 2012;33(6):361-371. Doi: 10.1159/000338606.

24. Almhdawi KA, Mathiowetz VG, White M, delMas RC. Efficacy of Occupational Therapy Task-oriented Approach in Upper Extremity Post-stroke Rehabilitation. Occup Ther Int 2016;23(4):444-456. Doi: 10.1002/oti.1447.

25. Yesavage JA, Brink TL, Rose TL, et al. Development and validation of a geriatric depression screening scale: a preliminary report. J Psychiatr Res 1982;17(1):37-49. Doi: 10.1016/0022-3956(82)90033-4.

26. Pachana NA, Byrne GJ, Siddle H, Koloski N, Harley E, Arnold E. Development and validation of the Geriatric Anxiety Inventory. Int Psychogeriatr 2007;19(1):103-114. Doi: 10.1017/S1041610206003504.

27. Rebok GW, Ball K, Guey LT, et al. Ten Year Effects of the Advanced Cognitive Training for Independent and Vital Elderly Cognitive Training Trial on Cognition and Everyday Functioning in Older Adults. J Am Geriatr Soc 2014;62(1):16-24. Doi: 10.1111/jgs.12607.

28. Singh MAF, Gates N, Saigal N, et al. The Study of Mental and Resistance Training (SMART) Study—Resistance Training and/or Cognitive Training in Mild Cognitive Impairment: A Randomized, Double-Blind, Double-Sham Controlled Trial. J Am Med Dir Assoc 2014;15(12):873-880. Doi: 10.1016/j.jamda.2014.09.010.

29. Optale G, Urgesi C, Busato V, et al. Controlling Memory Impairment in Elderly Adults Using Virtual Reality Memory Training: A Randomized Controlled Pilot Study. Neurorehabil Neural Repair 2009;24(4):348-357. Doi: 10.1177/1545968309353328.

30. Rozzini L, Costardi D, Chilovi BV, et al. Efficacy of cognitive rehabilitation in patients with mild cognitive impairment treated with cholinesterase inhibitors. Int J Geriatr Psychiatry 2007;22(4):356-360. Doi: 10.1002/gps.1681.

31. Gold DA. An examination of instrumental activities of daily living assessment in older adults and mild cognitive impairment. J Clin Exp Neuropsychol 2012;34(1):11-34. Doi: 10.1080/13803395.2011.614598.

32. Amunts J, Camilleri JA, Eickhoff SB, Heim S, Weis S. Executive functions predict verbal fluency scores in healthy participants. Sci Rep 2020;10:8549. Doi: 10.1038/s41598-020-65525-9.

33. Altuntaş O, Değerli MNÖ, Temizkan E, Ekici G. Psychometric properties of the Canadian occupational performance measure in older individuals. Turk J Med Sci 2024;54(1):338-347. Doi: 10.55730/1300-0144.5796.

34. de Waal MWM, Haaksma ML, Doornebosch AJ, Meijs R, Achterberg WP. Systematic review of measurement properties of the Canadian Occupational Performance Measure in geriatric rehabilitation. Eur Geriatr Med 2022;13(6):1281-1298. Doi: 10.1007/s41999-022-00692-8.

35. Brum PS, Borella E, Carretti B, Yassuda MS. Verbal working memory training in older adults: an investigation of dose response. Aging Ment Health 2020;24(1):81-91. Doi: 10.1080/13607863.2018.1531372.

36. Engvig A, Fjell AM, Westlye LT, et al. Effects of Cognitive Training on Gray Matter Volumes in Memory Clinic Patients with Subjective Memory Impairment. J Alzheimers Dis 2014;41(3):779-791. Doi: 10.3233/JAD-131889.

37. Hou J, Jiang T, Fu J, et al. The Long-Term Efficacy of Working Memory Training in Healthy Older Adults: A Systematic Review and Meta-Analysis of 22 Randomized Controlled Trials. J Gerontol B Psychol Sci Soc Sci 2020;75(8):1733-1748. Doi: 10.1093/geronb/gbaa077.

38. Fisher M, Holland C, Subramaniam K, Vinogradov S. Neuroplasticity-Based Cognitive Training in Schizophrenia: An Interim Report on the Effects 6 Months Later. Schizophr Bull 2010;36(4):869-879. Doi: 10.1093/schbul/sbn170.

39. Gates NJ, Vernooij RW, Di Nisio M, et al. Computerised cognitive training for preventing dementia in people with mild cognitive impairment. Cochrane Database Syst Rev 2019;2019(3). Doi: 10.1002/14651858.CD012279.pub2.

40. Fang P, Gao Y, Li Y, et al. Effects of computerized working memory training on neuroplasticity in healthy individuals: A combined neuroimaging and neurotransmitter study. NeuroImage 2024;296:120785. Doi: 10.1016/j.neuroimage.2024.120785.

41. Draganski B, Gaser C, Busch V, Schuierer G, Bogdahn U, May A. Changes in grey matter induced by training. Nature 2004;427(6972):311-312. Doi: 10.1038/427311a.

42. Sagi Y, Tavor I, Hofstetter S, Tzur-Moryosef S, Blumenfeld-Katzir T, Assaf Y. Learning in the Fast Lane: New Insights into Neuroplasticity. Neuron 2012;73(6):1195-1203. Doi: 10.1016/j.neuron.2012.01.025.

43. Strenziok M, Parasuraman R, Clarke E, et al. Neurocognitive enhancement in older adults: comparison of three cognitive training tasks to test a hypothesis of training transfer in brain connectivity. NeuroImage 2014;85(Pt 3):1027-1039. Doi: 10.1016/j.neuroimage.2013.07.069.

44. Rosen AC, Sugiura L, Kramer JH, Whitfield-Gabrieli S, Gabrieli JD. Cognitive Training Changes Hippocampal Function in Mild Cognitive Impairment: A Pilot Study. J Alzheimers Dis 2011;26(s3):349-357. Doi: 10.3233/JAD-2011-0009.

45. Kirchhoff BA, Anderson BA, Smith SE, Barch DM, Jacoby LL. Cognitive training-related changes in hippocampal activity associated with recollection in older adults. NeuroImage 2012;62(3):1956-1964. Doi: 10.1016/j.neuroimage.2012.06.017.

46. Lu Q, Wang Y, Qu B, et al. Structural and Functional Projections of the Nucleus Basalis of Meynert and Their Changes After Cognitive Training in Individuals With Mild Cognitive Impairment. CNS Neurosci Ther 2024;30(12):e14710. Doi: 10.1111/cns.70194.

FIGURE 1 (Descriptive figure legends)

Fig. 1 + p < 0.05 indicates a significant difference between the groups. ***** P < 0.05 indicates a significant improvement in each group, compared with the baseline values. **A** 20h of digital cognitive training group improved over time in COPM performance (p=0.001), compared to 10h of digital cognitive training group (p=0.97). After 3 months there was significant improvement in COPM performance of 20h DCT group (p=0.001) compare to 10h of DCT group (p=0.76). This effect remains in the 6 months assessment to 20h of DCT group (p=0.006). **B** 20h of digital cognitive training group improved over time in COPM satisfaction (p=0.002), compared to 10h of digital cognitive training group (p=0.34). After 3 months, there was significant improvement over time in COPM satisfaction (p<0.001) and this effect of time remains in 6 months assessment (p=0.008). **C** processing speed scores decreased significantly over time for the both groups post training: 10 h DCT group (p =0.001) and 20h DCT group (p = 0.04). After 3 months there was significant improvement over time in 10h of DCT group (p=0.02) and 20h of DCT group (p=0.001). In 6 months assessment follow up the both groups performed a significant effect over time 10h of DCT group (p=0.01) and 20h of DCT group (p=0.02). **D** Inhibitory control scores decreased significantly over time for 10h of DCT group (0.003) and no significant effect to 20h of DCT group(p=0.42). After 3 months the both groups showed a trend effect over time of 10h of DCT (p=0.07) and a significant effect over time of 10h of DCT (p=0.02). In 6 months follow-up assessment both 10h of DCT group decreased over time (p=0.02) and 20h of DCT group (p=0.003). Score = COPM, Canadian Occupational Performance Measure.

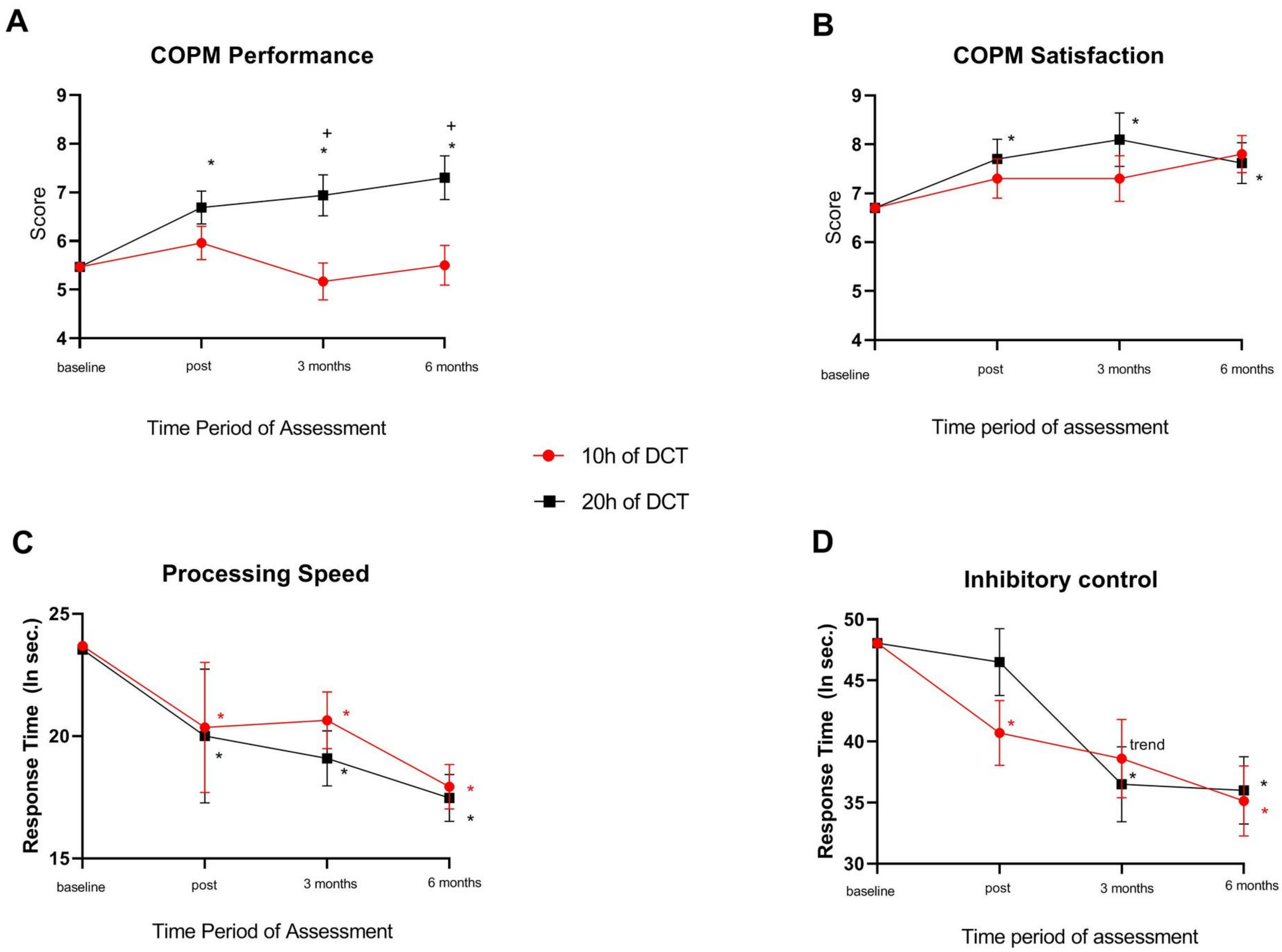
A
COPM Performance
Score
baseline
post
3 months
6 months
Time Period of Assessment
B
COPM Satisfaction
Score
baseline
post
3 months
6 months
Time period of assessment
10h of DCT
20h of DCT
C
Processing Speed
Response Time (In sec.)
baseline
post
3 months
6 months
Time Period of Assessment
D
Inhibitory control
Response Time (In sec.)
trend
baseline
post
3 months
6 months
Time period of assessment

Table 1. Participant characteristics

	Total (n=67)	10h of Digital Cognitive Training Group (n=34)	20h of Digital Cognitive Training Group (n=33)	p-value
Age, years	74.9(7.2)	75.4(7.7)	74.4(6.7)	0.57
Gender, %				0.56
Female	45(67.2)	23(67.6)	22(66.7)	
Male	22(32.8)	11(32.4)	11(33.3)	
Marital status, %				0.81
Single	8(11.9)	4(11.8)	4(12.1)	
Married	28(41.8)	13(38.2)	15(45.5)	
Divorced	9(13.4)	4(11.8)	5(15.2)	
Widowed	22(32.8)	13(38.2)	9(27.3)	
Education, years	14(5.2)	13.9(5.4)	14.1(5)	0.85
Physical activity, times per week	2(2.3)	2.02(2.2)	2(2.4)	0.79
Number of falls, past year	0.3(0.7)	0.3(0.9)	0.3(0.5)	0.70
Medications	4.3(2.9)	4.5(3.2)	4.1(2.5)	0.60
Previous contact with computer, %				0.83
None	12(18.8)	6(18.2)	6(19.4)	
Low	30(46.9)	16(48.5)	14(45.2)	
Medium	14(21.9)	6(18.2)	8(25.8)	
High	8(12.5)	5(15.2)	3(9.7)	
MoCA score	20.6(2.2)	20.4(2.2)	20.8(2.2)	0.51
GDS score	3.5(2.3)	3.3(2.2)	3.7(2.4)	0.40
GAI score	5.8(4.7)	5.4(4.9)	6.2(4.5)	0.52

Results are given as mean (standard deviation). MoCA: Montreal cognitive assessment. GDS: Geriatric Depression Scale. GAI: Geriatric Anxiety Inventory.

Table 2. Dose effect of 10 and 20 hours of digital cognitive training

Measurements (score range)	10h of Digital Cognitive Training Group (n=34)		20h of Digital Cognitive Training Group (n=33)		Main effect of time F (p)	Interaction Group*Time F (p)	Cohen`s d
Cognition	Baseline	10h Digital Cognitive Training	Baseline	20h Digital Cognitive Training			
MoCA (0-30)	20.61 (1.7)	22.51 (3.45)	20.67 (2.27)	22.87 (3.35)	0.71 (0.4)	0.10 (0.62)	0.08
Processing speed, seconds	24.3 (9.1)	20.4 (5.8)	22 (7.1)	20 (6.3)	**17.96 (0.00)***	0.01 (0.91)	0.00
Inhibitory control, seconds	48.8 (20.8)	40.7 (14.6)	47.2 (14.8)	46.5 (19.7)	**7.12 (0.01)***	*2.89 (0.09)*	0.04
Long term memory (0-15)	5.83 (2.8)	5.91 (3.3)	5.18 (2.8)	5.96(3.2)	*2.77 (0.09)*	0.56 (0.47)	0.22
Short term memory (0-15)	5.6 (2.9)	5.9 (3.2)	5.4 (2.9)	5.7(2.8)	**5.54 (0.02)**	0.01 (0.93)	0.00
Recognition (0-15)	10.8 (3.6)	11.7 (2.1)	11.1 (2.7)	11.59(2.6)	**34.2 (000)***	0.37 (0.54)	0.17
Learning over trials (0-70)	12.6 (4.9)	8.71 (4.7)	10.5 (7.8)	11.1 (4.09)	**25.67 (000)***	2.68 (0.11)	0.60
Verbal fluency, number of words	14.5 (3.4)	15.5 (4.6)	15 (3.7)	15.6 (3.9)	**7.05 (0.01)***	0.04 (0.83)	0.67
Functionality							
COPM, performance (1-10)	5.5 (1.6)	5.9 (2.3)	5.4 (2.2)	6.6 (1.8)	**5.86 (0.01)***	2.09 (0.15)	0.39
COPM, satisfaction (1-10)	6.6 (2.9)	7.3 (2.6)	6.8 (2.5)	7.4 (2.4)	**7.2 (0.01)***	0.003 (0.91)	0.00
TADQL (0-100)	14.5 (9.2)	14.3 (13.2)	15.8 (9.4)	11. 1(9.9)	2.41 (0.12)	*2.83 (0.09)*	2.25
Neuropsychiatric symptoms							
GDS (0-15)	3.3 (2.2)	2.8 (1.8)	3.8(1.8)	3.1(1.8)	**6.83 (0.01)***	0.001 (0.97)	0.00
GAI (0-20)	5 (4.8)	3.89 (4)	6.21(4.4)	3.71(4)	0.005 (0.95)	1.02 (0.31)	0.02

Results are given as mean (standard deviation). MoCA: Montreal cognitive assessment. COPM: Canadian Occupational Performance Measure. TADLQ: Technology Activities of Daily Living Questionnaire. GDS: Geriatric Depression Scale. GAI: Geriatric Anxiety Inventory.Bold represents statistically significant results ($p < 0.05$). Those marked with * are P values < 0.05, indicating a post hoc significant effect over time for each group (compared with the baseline value). Italics represent trends toward statistical significance ($0.05<P< 0.09$).

Table 3. Effect of intervention on cognitive domains and functionality after follow-up assessment (after 3 months).

Measurements (score range)	10h of Digital Cognitive Training Group (n=21)		20h of Digital Cognitive Training Group (n=20)		Main effect of time F (p)	Interaction Group* Time F (p)	Cohen`s d
Cognition	Baseline	10h Digital Cognitive Training	Baseline	20h Digital Cognitive Training			
MoCA (0-30)	20.77 (1.9)	21.76 (3.4)	20.47 (3.4)	21.76 (3.2)	3.12 (0.08)	0.01 (0.81)	0.00
Processing speed, seconds	26.2 (9.5)	20.8 (5.7)	21.3 (6.4)	18.9 (4.8)	**16.4 (000)***	0.18 (0.66)	0.14
Inhibitory control, seconds	42.7 (15.8)	38.6 (12.5)	48.2 (15.8)	35.8 (11.9)	**2.93 (0.007*)**	2.46 (0.12)	0.52
Long term memory (0-15)	6.04 (2.9)	6.1 (3.9)	5.2 (3.1)	5.1 (3.8)	0.22 (0.6)	0.13 (0.72)	0.12
Short term memory (0-15)	6.1 (2.7)	6.1 (3.8)	5.6 (3.1)	6.01 (3.7)	1.01 (0.3)	0.83 (0.77)	0.08
Recognition (0-15)	11.09 (2.9)	12.42 (1.9)	10.8 (3.03)	11 (3.4)	**13.74 (0.001)***	3.13 (0.08)	0.58
Learning over trials (0-70)	12.65 (4.9)	12.29 (6.5)	10.56 (7.8)	11.06 (6.6)	**14.28 (0.001)***	0.15 (0.64)	0.14
Verbal fluency, number of words	15.5 (3.7)	16.2 (4.4)	16 (4.1)	15.4 (4.1)	3.3 (0.07)	0.79 (0.37)	0.28
Functionality							
COPM, performance (1-10)	5 (1.6)	5.1 (1.6)	5.5 (2.4)	6.9 (1.7)	**16.9 (0.00)***	**8.8 (0.005)**	1.03
COPM, satisfaction (1-10)	6.6 (2.9)	7.3 (2.5)	7.0 (2.5)	8.1 (1.7)	**13.3 (0.001)***	0.9 (0.34)	0.34
TADQL (0-100)	11.4 (6.3)	15.0	13.0 (13.1)	15.1 (14.5)	1.22 (0.27)	0.007 (0.93)	0.00
Neuropsychiatric symptoms							
GDS (0-15)	3.4(2.8)	3.8(2.3)	3.6(2.3)	3.4(1.6)	**9.97(0.003)***	0.64(0.42)	0.22
GAI (0-20)	4.7(4.5)	3.3(3.5)	6.4(4.5)	4.8(5.2)	0.13(0.7)	0.14(0.7)	0.10

Results are given as mean (standard deviation). MoCA: Montreal cognitive assessment. COPM: Canadian Occupational Performance Measure. TADLQ: Technology Activities of Daily Living Questionnaire. GDS: Geriatric Depression Scale. GAI: Geriatric Anxiety Inventory.Bold represents statistically significant results (p < 0.05). Those marked with * are P values <.05, indicating a post hoc significant effect over time for each group (compared with the baseline value). Italics represent trends toward statistical significance (0.05<P<0.09).

Table 4. Effect of intervention on cognitive domains and functionality after follow-up assessment (after 6 months).

Measurements (score range)	10h of Digital Cognitive Training Group (n=16)		20h of Digital Cognitive Training Group (n=15)		Main effect of time F (p)	Interaction Group* Time F (p)	Cohen`s d
Cognition	Baseline	10h Digital Cognitive Training	Baseline	20h Digital Cognitive Training			
MoCA (0-30)	20.62 (1.8)	21.93 (3.45)	20.76 (2.1)	23.47 (3.35)	2.72 (0.11)	1.64 (0.2)	0.47
Processing speed, seconds	25.8 (9)	17.9 (4.3)	21 (7.4)	18.3 (3.1)	**23.3 (0.000)***	2.46 (0.12)	0.062
Inhibitory control, seconds	42.8 (16.6)	34.3 (11.2)	47.1 (17.5)	36.2 (9.3)	**11.06 (0.003)***	0.05 (0.82)	0.08
Long term memory (0-15)	6.06 (3)	6.6 (3)	5.1 (3.2)	5.8 (3.7)	0.49 (0.48)	0.001 (0.9)	0.00
Short term memory (0-15)	6.06 (2.9)	5.5 (4)	5.53 (3.3)	6 (3.1)	2.22 (0.14)	0.39 (0.5)	0.02
Recognition (0-15)	11.6 (1.5)	12.46 (2.2)	10.3 (2.7)	11.93 (3.5)	**5.4 (0.02)***	0.007 (0.9)	0.00
Learning over trials (0-70)	12.65 (4.9)	5.24 (6)	10.56 (7.8)	7.8 (7.9)	0.03 (0.83)	1.88 (0.1)	0.50
Verbal fluency, number of words	15.7 (4.1)	16.1 (5.2)	15.7 (3.2)	16.1 (3.7)	0.006 (0.93)	0.001 (0.97)	0.00
Functionality							
COPM, performance (1-10)	5.3 (1.6)	5.5 (1.8)	5.4 (2.7)	7.3 (1.3)	**18.9 (0.000)***	**9.1 (0.006)**	1.18
COPM, satisfaction (1-10)	7.1 (2.7)	7.8 (2.2)	6.8 (2.5)	7.6 (2)	**8.7 (0.007)***	0.01 (0.89)	0.06
TADQL (0-100)	11.0 (6.1)	10.3 (6.9)	16.6 (9.6)	13.77 (8.8)	0.809 (0.37)	0.01 (0.91)	0.00
Neuropsychiatric symptoms							
GDS (0-15)	3.3(2.8)	4.1(2.7)	3.7(2.3)	4.2(1.6)	**8.1(0.007)***	0.33(0.86)	0.06
GAI (0-20)	4.5(4.5)	3.17(3.7)	6.9(4.5)	4.59(4.7)	0.27(0.67)	0.02(0.81)	0.06

Results are given as mean (standard deviation). MoCA: Montreal cognitive assessment. COPM: Canadian Occupational Performance Measure. TADLQ: Technology Activities of Daily Living Questionnaire. GDS: Geriatric Depression Scale. GAI: Geriatric Anxiety Inventory.Bold represents statistically significant results (p < 0.05). Those marked with * are P values <.05, indicating a post hoc significant effect over time for each group (compared with the baseline value). Italics represent

trends toward statistical significance ($0.05 < P < 0.09$).
